# Identification of putative interactions between swine and human influenza A virus nucleoprotein and human host proteins

**DOI:** 10.1186/s12985-014-0228-6

**Published:** 2014-12-30

**Authors:** Alex Generous, Molly Thorson, Jeff Barcus, Joseph Jacher, Marc Busch, Heidi Sleister

**Affiliations:** Biology Department, Drake University, 1344 27th St., Des Moines, IA 50311 USA

**Keywords:** Influenza A virus, Nucleoprotein, Yeast two-hybrid, Host restriction

## Abstract

**Background:**

Influenza A viruses (IAVs) are important pathogens that affect the health of humans and many additional animal species. IAVs are enveloped, negative single-stranded RNA viruses whose genome encodes at least ten proteins. The IAV nucleoprotein (NP) is a structural protein that associates with the viral RNA and is essential for virus replication. Understanding how IAVs interact with host proteins is essential for elucidating all of the required processes for viral replication, restrictions in species host range, and potential targets for antiviral therapies.

**Methods:**

In this study, the NP from a swine IAV was cloned into a yeast two-hybrid “bait” vector for expression of a yeast Gal4 binding domain (BD)-NP fusion protein. This “bait” was used to screen a Y2H human HeLa cell “prey” library which consisted of human proteins fused to the Gal4 protein’s activation domain (AD). The interaction of “bait” and “prey” proteins resulted in activation of reporter genes.

**Results:**

Seventeen positive bait-prey interactions were isolated in yeast. All of the “prey” isolated also interact in yeast with a NP “bait” cloned from a human IAV strain. Isolation and sequence analysis of the cDNAs encoding the human prey proteins revealed ten different human proteins. These host proteins are involved in various host cell processes and structures, including purine biosynthesis (PAICS), metabolism (ACOT13), proteasome (PA28B), DNA-binding (MSANTD3), cytoskeleton (CKAP5), potassium channel formation (KCTD9), zinc transporter function (SLC30A9), Na+/K+ ATPase function (ATP1B1), and RNA splicing (TRA2B).

**Conclusions:**

Ten human proteins were identified as interacting with IAV NP in a Y2H screen. Some of these human proteins were reported in previous screens aimed at elucidating host proteins relevant to specific viral life cycle processes such as replication. This study extends previous findings by suggesting a mechanism by which these host proteins associate with the IAV, i.e., physical interaction with NP. Furthermore, this study revealed novel host protein-NP interactions in yeast.

## Background

Influenza A viruses (IAVs) are important pathogens that affect the health of humans and many additional animal species. In humans, seasonal IAV infection presents as a non-fatal, uncomplicated, acute infection characterized by the presence of upper respiratory symptoms as well as fever, headache, soreness and fatigue lasting 2–5 days [1]. However, deaths from seasonal IAV infections often arise when the normal flu symptoms are exacerbated by compromised immunity or age [1]. In addition to seasonal influenza A viruses, pandemic strains periodically appear causing increased mortality rates. For example, the 1918 Spanish flu resulted in approximately 50 million human deaths [2]. The most recent pandemic resulted from the emergence of the swine-origin H1N1 virus [3,4]. Due to influenza A’s potential for mortality, high mutation rates (resulting in genetic drift) and pandemic potential (resulting from genetic reassortment), it is critical to learn more about the virus, especially as it pertains to virulence, transmissibility, and identification of potential targets for development of therapeutics.

Influenza A viruses exhibit a broad host range beyond humans [5]. Waterfowl serve as the central reservoir species. In addition to humans, various IAV subtypes circulate in pigs, poultry, horses, dogs as well as other species. Subtypes (H1N1 and H3N2) that circulate in the human population also circulate in the swine populations [6]. In addition to the concern for IAV in terms of swine health [7], IAV infection in swine is also important due to the potential for zoonotic infections as well as the potential for serving as a mixing vessel for the generation of human pandemic viruses [5]. The majority of inter-species transmission research has focused on amino acids in the hemagglutinin affecting viral attachment and entry [8]. Although known to be important, other than amino acids associated with temperature sensitivity (e.g., PB2 627 [9] or 701 [10]), the effects of differences in amino acid residues in proteins of the replication complex on species specificity is less well understood. Previous evidence suggests that there may be a role in some of these replication complex proteins in allowing for zoonotic infections [11].

The genome of the enveloped influenza A virion consists of eight segments of negative-sense single-stranded RNA that encode at least ten viral genes: Hemagglutinin (HA), Neuraminidase (NA), Matrix 2 protein (M2), Matrix 1 protein (M1), Non-structural Protein 1 (NS1), Non-structural Protein 2 (NS2), Nucleoprotein (NP), Polymerase Basic 1 (PB-1), Polymerase Basic 2 (PB-2), and Polymerase Acidic (PA) [1]. The viral envelope contains HA, NA, and M2 proteins, and M1 proteins form a layer inside the envelope. The IAV RNA genome located inside the virion is coated with NP and is bound by the replication complex consisting of PB1, PB2, and PA. The NP, the focus of this study, encoded by the fifth IAV RNA segment, binds with high affinity to viral RNA [12]. NP plays a role in viral RNA replication and transcription [12]. More recent data suggests that NP binds the polymerase as well as the newly replicated RNA and may act as a processivity factor that is necessary for replication of the viral RNA to be completed [13]. Phylogenic analysis of IAV NP shows distinct lineages of NP based on the host species [14,15]. Within the NP, there are amino acid signatures found within different host species [16,17]. These host-specific amino acid residues may result in differences in affinities for the various host proteins with which they interact (e.g., importin α1 [18], F- actin [19], nuclear factor 90 [20], cyclophilin E [21], exportin 1 [22], HMGB1 [23], HMGB2 [23], MxA/Mx1 [24,25], HSP40 [26], karyopherin alpha [27,28], clusterin [29], Raf-2p48/BAT1/UAP56 [30], Aly/REF [31], Tat-SF1 [32], TRIM22 [33], and alpha actinin 4 [34]) or they may result in differences in how the NP interacts with other viral proteins that have also made host-specific adaptations [11]. Given the suggestion that NP plays a role in determining host range [5,35-37], it is important to identify all host proteins that interact with NP.

Replication of the IAV is dependent on the host cell machinery and interactions between host proteins and viral proteins. Therefore any inquiry attempting to investigate the life cycle of IAV must incorporate host-pathogen protein interactions in order to truly provide a mechanistic understanding of the process. Previous screens have identified important protein-protein interactions between IAV and host proteins, but often results are not consistent between screens. Therefore, corroborating evidence with additional screens is crucial to accumulating the best understanding of critical pathogen-host interactions in viral replication. As examples, several genome-wide screens have been performed using RNAi to systematically knockdown host genes and evaluate the effect on various stages of the IAV life cycle [38-41]. An integrated approach carried out by Shapira and colleagues involved transcription profiling and yeast two hybrid screens using specific viral proteins as baits [28]. Proteomics approaches have revealed host proteins found within viral particles [42,43]. The Random Homozygous Gene Perturbation strategy was used to identify host factors that prevent influenza-mediated killing of host cells [44].

In order to better understand the role of NP-host protein interactions in IAV replication, a Gal4-based yeast two-hybrid (Y2H) assay was used in this study. The Y2H system allows for the identification of unique human binding partners with NP. A “bait” plasmid encoding the binding domain (BD) of the Gal4 transcriptional activator fused to NP and a “prey” plasmid encoding Gal4’s activation domain (AD) fused to a protein encoded by a human cDNA are introduced into a yeast strain containing Gal4-responsive reporter genes. Interaction of the bait and prey brings together Gal4’s BD and AD, resulting in transcriptional activation of reporter genes [45]. The Y2H approach has successfully identified cellular factors (e.g., Raf-2p48/BAT1/UAP56, Hsp40, KPNA1, KNPA3, KPNA6, C16orf45, GMCL1, MAGED1, MLH1, USHBP1, ZBTB25, CLU, Aly/REF, and ACTN4) that interact with the IAV NP [26-31,34], (Table 1). In this study, the nucleoprotein from a classical swine H1N1 IAV (Sw/NC/44173/00) was used as the “bait” in a Y2H screen against a “prey” HeLa cDNA library. To investigate if the origin of NP affects the ability of the interactions to take place, the nucleoprotein from a contemporary human H3N2 IAV (A/Ca/07/04) was also used as the “bait” in a Y2H screen against the same prey HeLa cDNA library. By determination of the putative interaction partners between NP and human proteins, the possible functions of the newfound protein-protein interactions or verification and perhaps elucidation of previously identified host factors may be further investigated.

**Table 1:** **Summary of identified bait-prey interactions and previous reports of host protein association with influenza A virus**

**Gene**	**Protein description**	**Complete ORF**	**Number of prey clones**	**Prey clones**	**Previous reports with IAV**
PAICS	Phosphoribosylaminoimidazole carboxylase	Yes	3	R3, R9, R15	Karlas et al. [40], Kumar and Nanduri [47], Kroeker et al. [48]
MSANTD3	Myb/SANT-like DNA-binding domain containing 3	No	3	R10, R13, R27	N/A
FLJ30306	PREDICTED: Homo sapiens uncharacterized LOC101059922, mRNA	Yes	2	R23, R33	N/A
PA28B	Proteasome activator subunit 2	Yes	2	R6, R19	N/A
KCTD9	Potassium channel tetramerisation domain containing 9	No	2	R34, R36	N/A
ACOT13	Acyl-CoA thioesterase 13	Yes	1	R11	N/A
TRA2B	Transformer 2 beta homolog (Drosophila)	Yes	1	R12	Zhu et al. [56]
SLC30A9	Solute carrier family 30 (zinc transporter), member 9	No	1	R28	Kumar and Nanduri [47], Sui et al. [44]
ATP1B1	ATPase, Na+/K+ transporting, beta 1 polypeptide	No	1	R7	Mi et al. [51], Liu et al. [50]
CKAP5	Cytoskeleton associated protein 5	No	1	R29	N/A
**Host proteins identified as interacting with IAV nucleoprotein in previously published yeast two-hybrid screens**					
BAT1/UAP56	RNA-dependent ATPase	N/A	N/A	N/A	Momose et al. [30]
HSP40	Heat shock protein 40	N/A	N/A	N/A	Sharma et al. [26]
NPI-1/ SRP1/KPNA1	Karyopherin α1	N/A	N/A	N/A	O’Neill and Palese [27], Shapira et al. [28]
KPNA3	Karyopherin α3	N/A	N/A	N/A	Shapira et al. [28]
KPNA6	Karyopherin α6	N/A	N/A	N/A	Shapira et al. [28]
C16orf45	-	N/A	N/A	N/A	Shapira et al. [28]
GMCL1	Germ cell-less, spermatogenesis associated 1	N/A	N/A	N/A	Shapira et al. [28]
MAGED1	Melanoma antigen family D.1	N/A	N/A	N/A	Shapira et al. [28]
MLH1	MutL homolog 1	N/A	N/A	N/A	Shapira et al. [28]
USHBP1	Usher syndrome 1C binding protein 1	N/A	N/A	N/A	Shapira et al. [28]
ZBTB25	Zinc finger and BTB domain containing 25	N/A	N/A	N/A	Shapira et al. [28]
CLU	Clusterin	N/A	N/A	N/A	Tripathi et al. [29]
ALY/REF	RNA export adaptor protein	N/A	N/A	N/A	Balasubramaniam et al. [31]
ACTN4	Alpha-actinin 4	N/A	N/A	N/A	Sharma et al. [34]

Reported herein are ten potential human host cell proteins that interact with IAV NP in a Y2H screen. The relative strengths of these protein-protein interactions were characterized by a plating assay and beta-galactosidase assay.

## Results

### Constructing an NP bait and screening a human cDNA prey library

**Figure 1:**
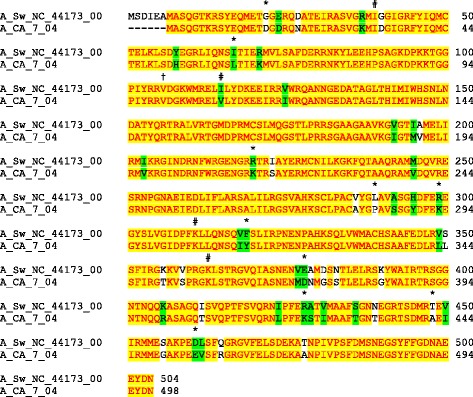
**Sequence alignment of nucleoproteins from swine and human influenza A virus strains.** The swine and human NPs are 89.1% identical (yellow highlighting) and 94.8% similar (yellow and green highlighting). Nonconservative amino acids differences are represented in white. Amino acids with * represent signature amino acids associated with avian influenza A NP, and # and † represent signature amino acids associated with avian influenza A NP based on Chen et al. [16] and Pan et al. [17]. † Denotes the only deviation of A/Sw/NC 44173/00 from the consensus swine residues based on Pan et al. [17].

The nucleoprotein open reading frame from the classical swine IAV H1N1 strain A/Sw/NC/44173/00 and contemporary human IAV H3N2 strain A/Ca/07/04 encode proteins with 89.1% identity and 94.8% similarity (Figure 1). Of the 49 amino acids that differ between the swine and human NPs, 29 are conservative differences. However, it is worth noting that several of these amino acid differences are signatures that differ between avian, swine and human isolates [16,17]. The two NP ORFs were amplified by PCR, and the resulting PCR products were separately inserted by recombination cloning into Y2H bait vector pGBKT7 as a C-terminal fusion with Gal4p’s DNA binding domain. Correct constructs were confirmed by DNA sequence analysis. Gal4(BD)-NP fusion proteins isolated from yeast cells were detected as 77 KDa bands on a Western blot probed with an anti-Myc antibody (data not shown). When introduced alone into yeast Y2HGold cells, the swine and human NP baits constructs were not toxic to yeast and did not autoactivate the Y2H reporter genes, indicating they were suitable for use in a Y2H screen (data not shown).

Using a Y2H assay with Gal4 (BD)-swine NP as a bait and a human HeLa cDNA prey library, approximately 2 × 10^6^ clones were screened resulting in 17 positive bait-prey interacting yeast strains. Sequence and bioinformatics analysis of the prey plasmids indicated that these 17 prey plasmids, listed by their initial positive interaction identification number (R3, R6, etc.), represent ten different human cDNAs (Table 1). An independent screen using Gal4 (BD)-human NP as a bait and a human HeLa cDNA prey library yielded a subset of these same host protein targets. Some of the identified clones contain partial cDNAs. Three independent prey plasmids isolated contained human cDNAs phosphoribosylaminoimidazole carboxylase, phosphoribosylaminoimidazole succinocarboxamide synthetase (PAICS) and another three contained Myb/SANT-like DNA-binding domain containing 3 (MSANTD3). Two independent prey plasmids were isolated for each of three human cDNAs: proteasome activator subunit 2 (PA28B), potassium channel tetramerisation domain (KCTD9), and an uncharacterized protein (FLJ30306). Each of the remaining five prey plasmids contained different human cDNAs.

### Evaluation of the strength of bait-prey interactions

**Figure 2:**
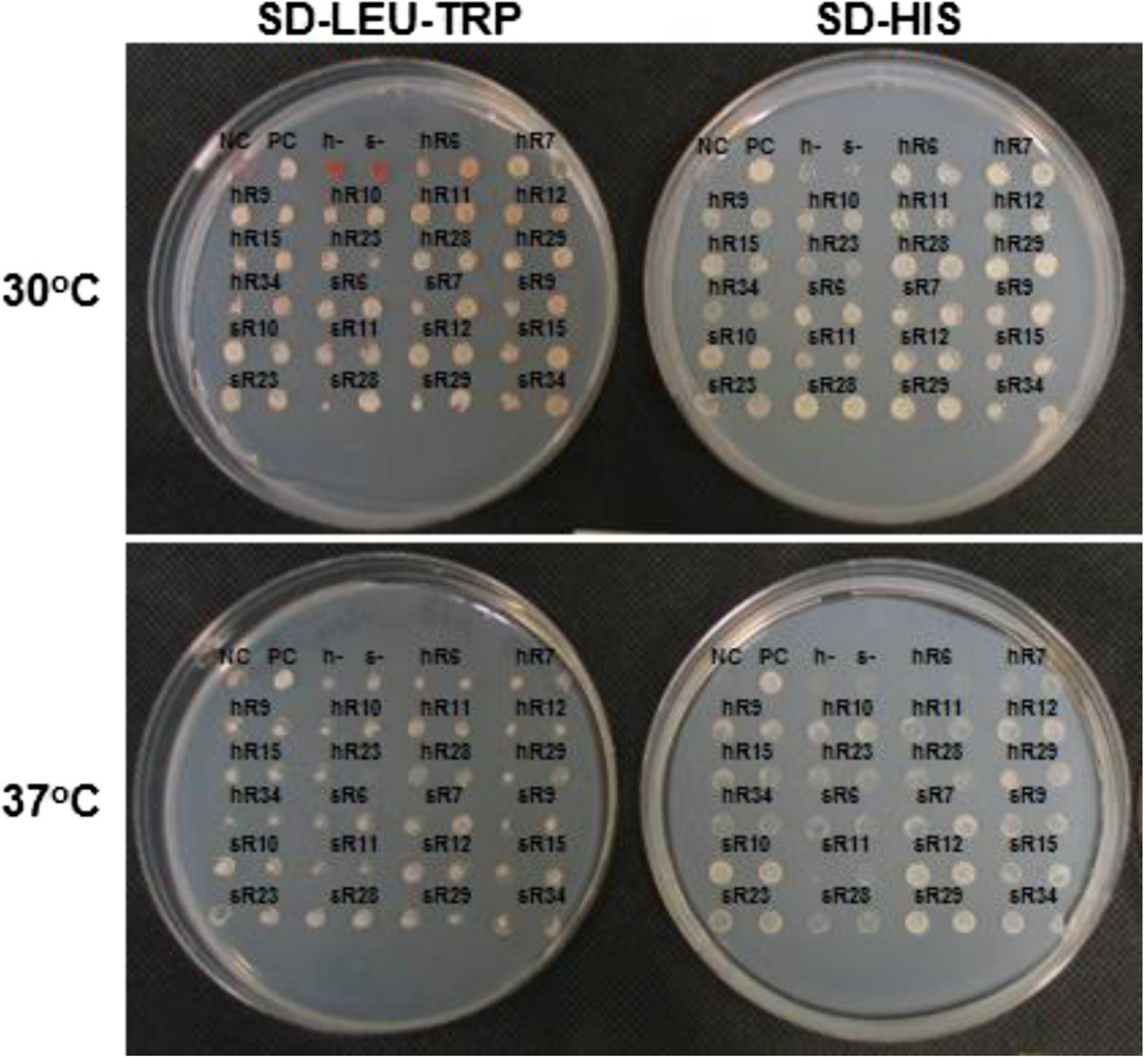
**Maintenance of protein-protein interactions at 30°C and 37°C.** Yeast cells containing both a bait plasmid and prey plasmid were spotted in duplicate onto agar plates and incubated at 30°C or 37°C. SD-Leu-Trp media serves as a growth control, and SD-His is protein interaction reporter media. Growth on SD-His is represented as normal growth (++), reduced growth (+), and no growth (−) (see Table 2). NC and PC represent negative and positive controls, respectively. Additional yeast strain negative controls include h- (human NP bait and empty prey vector) and s- (swine NP bait and empty prey vector).

The strength of interaction between each NP bait (swine or human) and human prey in yeast was measured using a qualitative growth assay and a quantitative β-galactosidase activity assay. First, yeast growth on selective media after cotransformation with swine or human NP and one of the identified prey was tested at both 30°C and 37°C as a qualitative metric of the protein-protein interaction’s strength (Figure 2, Table 2). All strains, including the positive and negative controls, grew on SD-Leu-Trp which selects for the presence of both bait and prey plasmids. Most strains showed decreased growth at 37°C relative to the more permissive temperature 30°C. Strains examined at 37°C which contained either swine or human NP bait grew best in the presence of prey CKAP5, MSANTD3, or TRA2B. In contrast, at 37°C strains containing swine NP grew poorly with prey ACOT13, whereas strains containing human NP grew poorly with prey PA28B.

**Table 2:** **Summary of affinity of bait-prey interactions**

			Growth on SD-His agar plates*				β-Gal activity**	
**Gene**	**Protein description**	**Prey clone**	**Human (30°C)**	**Human (37°C)**	**Swine (30°C)**	**Swine (37°C)**	**Human NP**	**Swine NP**
PAICS	Phosphoribosylaminoimidazole carboxylase	R15	(++)	(+)	(++)	(++)	2.2	30.4
MSANTD3	Myb/SANT-like DNA-binding domain containing 3	R10	(++)	(++)	(++)	(++)	13.9	18.2
FLJ30306	PREDICTED: Homo sapiens uncharacterized LOC101059922, mRNA	R23	(+)	(+)	(++)	(++)	1.9	5.4
PA28B	Proteasome (prosome, macropain) activator subunit 2	R6	(++)	(−)	(++)	(+)	6.7	33.3
KCTD9	Potassium channel tetramerisation domain containing 9	R34	(+)	(+)	(++)	(++)	5.0	16.4
ACOT13	Acyl-CoA thioesterase 13	R11	(++)	(+)	(++)	(−)	6.9	15.5
TRA2B	Transformer 2 beta homolog (Drosophila)	R12	(++)	(++)	(++)	(++)	1.9	0.85
SLC30A9	Solute carrier family 30 (zinc transporter), member 9	R28	(++)	(+)	(++)	(+)	82.9	119.9
ATP1B1	ATPase, Na+/K+ transporting, beta 1 polypeptide	R7	(++)	(+)	(++)	(++)	3.4	4.3
CKAP5	Cytoskeleton associated protein 5	R29	(++)	(++)	(++)	(++)	70.1	90.3

* Results from growth on SD-His protein interaction assay media are presented as: (++) normal growth, (+) reduced growth, and (-) no growth (Figure 2).

**Figure 3:**
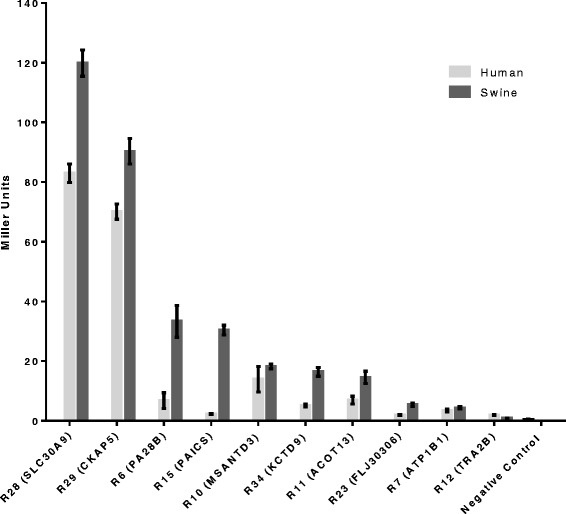
**β-galactosidase activity in liquid culture.** Β-gal activity is a quantitative measure of the strength of interaction between bait swine or human NP and prey human host proteins identified in the Y2H screen. The β-gal activity for each interacting bait-prey pair is expressed in Miller units and represents the mean and standard error of the mean of at least eight independent samples.

** Mean β-galalactosidase activity from at least eight replicates reported in Miller units (Figure 3).

The strength of the interactions between IAV NP and host proteins identified by the library screen was also investigated by measuring the β-gal activity in liquid yeast cultures (Figure 3, Table 2). As observed with the qualitative growth assay, for all ten prey proteins, interactions were observed with both the swine and human NP. Of the ten interactions investigated, yeast strains containing swine or human NP and human prey SLC30A9 and CKAP3 showed the highest levels of β-gal activity compared to the other prey proteins examined. This is consistent with the growth of yeast strains containing human NP and SLC30A9 and CKAP3 on selective media at 37°C ( Figure 2, Table 2). Even strains with the lowest levels of β-gal activity observed (TRA2B and ATP1B1) have double the β-gal activity of the negative control. While the data suggest there may be differences in the strength of bait-prey interactions based on NP host origin, these differences should be interpreted carefully given the nature of the assay.

## Discussion

This Y2H screen identified multiple candidate host proteins that were previously identified in genome-wide screens as playing a role in the IAV infection cycle (PAICS, ATP1B1, SLC30A9, and TRA2B), while also identifying new potential interactors with IAV nucleoprotein (ACOT13, CKAP5, KCTD9, PA28B, MSANDTD3, and FLJ30306). These results complement previous reports by suggesting the mechanism for involvement of the host proteins with the IAV (i.e. interaction with the NP). The yeast screen is internally validated by the observation that half of the prey proteins identified were independently isolated multiple times during the screen. All host prey proteins identified interact in yeast with IAV NP from both human and swine isolates. Although there can be variability in gene expression among yeast colonies containing the same combination of bait/prey, both a qualitative growth assay and a quantitative β-gal assay yielded similar results in terms of the strength of interaction between IAV NP bait and human prey proteins. Notably, CKAP5 displayed good growth at the more stringent 37°C in the qualitative assay and also had high β-gal activity. The putative interacting host proteins represent a variety of cellular processes, consistent with the diversity of processes required during the IAV infection cycle.

Phosphoribosylaminoimidazole carboxylase, phosphoribosylaminoimidazole succinocarboxamide synthetase (PAICS) is an enzyme necessary to catalyze the sixth and seventh steps of the *de novo* purine biosynthesis pathway [46]. PAICS has been identified by two separate RNAi screens as being crucial to IAV replication [40,47]. Furthermore, proteins involved in purine biosynthesis, including PAICS, were found to be significantly up-regulated during an IAV infection [48]. The data presented herein suggest that the requirement for PAICS during IAV infection may be through the interaction with NP.

The sodium/potassium-transporting ATPase subunit beta-1 (ATP1B1) represents another host protein that interacts in yeast with NP and that has been previously shown to be important for influenza virus replication in host cells. It belongs to the family of Na+/K + −ATPase beta chain proteins. Na+/K + −ATPase is necessary for the creation and maintenance of electrochemical gradients of sodium and potassium ions on either side of the plasma membrane [49]. This gradient is used by cells to permit functional nervous and muscular excitability, osmoregulation, and active transport of numerous molecules [49]. The beta subunit functions to regulate the quantity of sodium pumps transported to the plasma membrane [49]. Using a quantitative proteomics approach, ATP1B1 was one of 43 proteins found to be significantly up-regulated in IAV-infected primary human alveolar macrophages [50]. In a Y2H study, Mi and colleagues [51] isolated ATP1B1 as a protein that binds the cytoplasmic domain of IAV M2 and subsequently showed that knockdown of ATP1B1 in MDCK cells suppressed IAV replication [51]. These studies suggest that ATP1B1 may be interacting with multiple IAV proteins.

The solute carrier family 30 (zinc transporter), member 9 (SLC30A9) is involved in nucleotide-excision repair of human DNA [52] and has an efflux motif characteristic of proteins in the SLC30 zinc efflux transporter family [53]. SLC30A9 protein found in the cytoplasm can bind nuclear receptors and/or nuclear receptor coactivators and translocate to the nucleus where it regulates gene transcription [54]. SLC30A9 was identified as a possible host target that confers upon the host cell resistance to IAV infection [44]. How SLC30A9 might interact with NP and be necessary for IAV replication is unclear.

Another protein identified by this screen is TRA2B, a tissue-specific splicing factor [55]. TRA2B protein binds to AGAA and CAA RNA sequences affecting the inclusion of introns in the processed transcript [55]. With respect to influenza, expression of TRA2B protein was reported to be down-regulated in porcine alveolar macrophage cells infected by two swine IAVs [56]. Because influenza genes M2 and NS2 are spliced, it is possible that TRA2B is important for the splicing of these genes. However, how NP might be involved in TRA2B’s function in IAV replication is unknown.

Another binding partner identified is Acyl CoA thioesterase 13 (ACOT13), a eukaryotic protein also known as thioesterase superfamily member 2 (Them2) [57,58]. ACOT13 catalyzes fatty acyl-CoA hydrolysis that exists primarily in association with mitochondria [57]. ACOT13/Them2 was identified in a Y2H screen focused on phosphatidylcholine transfer protein, suggestive of a role in fatty acid metabolism [57]. Although ACOT13 has not previously been reported to play a role in the IAV life cycle, another member of the ACOT family (ACOT9) which has two hot dog fold domains (ACOT13 has one) [59] has been shown to physically interact with the IAV PA protein [28]. How ACOT13 may be interacting with NP during IAV replication is unclear.

Cytoskeletal associated protein 5 (CKAP5, also known as TOG) binds microtubules and is important for the process of centrosomal microtubule assembly especially during mitosis [60]. The microtubule network of the cell plays a role in movement of materials throughout the cell, including during infection [61]. TOG2 binds to a ribonucleoprotein and plays a role in RNA trafficking [62]. While a specific role for CKAP5 in the IAV life cycle is unknown, a genome-wide siRNA screen revealed CKAP5 as one of 96 human genes that supports replication of another RNA virus- the hepatitis C virus [63]. It’s possible that CKAP5 functions during the IAV infection cycle to organize microtubules for trafficking of viral components.

Another interactor identified in this screen, KCTD9, has been previously shown to interact with a subunit of the Mediator complex, acting as a scaffold between regulatory proteins and the RNA polymerase II [64]. In addition, KCTD9 homolog FIP2 acts as a cytoskeletal rearrangement protein suspected to be involved in nuclear export in some plants through the organization of actin cables [64]. Perhaps KCTD9 aids in translocating NP to the nucleus for replication and transcription of the viral RNA. It has been shown that the translocation of NF-E2 related factor 2 (Nrf2) is mediated by cytoskeletal rearrangement in the oxidative stress response [65], and the level of Nrf2 expression is associated with IAV entry and replication in nasal epithelial cells [66].

Also identified by this screen was PA28B, a protein associated with the proteasome complex. In the typical proteasome, there is a 19S regulator, which is replaced by the 11S regulator in the modified immunoproteasome [67]. PA28B is part of the proteasome complex and acts as a subunit in the 11S alternate regulator. It is referred to as the beta subunit and is comprised of three beta and three alpha subunits arranged in a heterohexameric ring [68]. The proteasome, as a whole, is used for the cleaving of peptides and the 11S regulator, in particular, induces the degradation of short peptides, but not entire proteins [68]. Induction of 11S regulator expression is the responsibility of interferon gamma. PA28B is involved in cleavage of the peptides which bind to the major histocompatibility complex (MHC). It is possible that NP interacts with PA28B to prevent MHC I presentation of IAV antigens.

Myb/SANT-like DNA-binding domain containing 3 (MSANTD3) is a member of the MSANTD3 family which contains DNA binding domains for Myb proteins and the SANT domain family. Myb proteins are proto-oncogenes which are necessary for hematopoiesis and possibly involved in tumorigenesis [69]. The SANT domain permits the interaction of chromatin remodeling proteins with histones and is found in chromatin-remodeling complexes as well as nuclear receptor co-repressors [70]. NP interactions with MSANTD3 could have an effect on gene transcription of the host through chromatin modification, presumably to inhibit antiviral genes or up-regulate genes beneficial to viral replication.

The screen also identified FLJ30306 as a potential interactor with NP. FLJ30306 maintains a sequence similarity to retroviral elements, resulting in its classification as endogenous retrovirus group K3, member 1 [*Homo sapiens*] [71]. The endogenous retrovirus sequence was revealed by sequence analysis of the mRNA, not by direct protein sequencing. It is unclear whether the protein carries out a normal cellular effect, such as endogenous retrovirus 1’s (ERV-1) use in placental syncytia formation or if it is a result of mis-regulation due to influenza infection [71]. In Hodgkin’s lymphoma it has been shown that many endogenous retroviruses are reactivated and produce virus-like particles [71]. It may be that instead of directly utilizing FLJ30306 influenza may simply be up-regulating its expression due to its pathogenesis. Alternatively, FLJ30306 may contain useful enzymatic activity for influenza replication through an unknown mechanism. A characterization of normal FLJ30306 function would allow for appropriately testing either hypothesis.

All human host proteins identified in this Y2H screen interact in yeast with NP from both swine and human IAV strains. While the data suggest there may be differences in the strength of bait-prey interactions in yeast based on NP host origin, these differences should be interpreted carefully given the nature of the assay. Given the suggestion that NP affects species specificity, additional screens using NP from other IAV strains as the bait and/or cDNA libraries from other susceptible species may shed light on amino acids within the NP and specific host factors that are responsible for host restrictions. As is the case with other published screens (e.g., [72]), it will be important to verify the putative interactions described here in infected cells by co-immunoprecipitation, co-localization, or RNA interference (e.g., [34]) and to investigate the role of the identified host proteins in the IAV life cycle.

## Conclusions

A Y2H screen using a classical swine influenza A virus nucleoprotein as a bait resulted in isolation of ten different putative interacting human host proteins involved in a variety of cellular processes and structures: purine biosynthesis (PAICS), metabolism (ACOT13), proteasome (PA28B), DNA-binding (MSANTD3), cytoskeleton (CKAP5), potassium channel formation (KCTD9), zinc transporter function (SLC30A9), Na+/K+ ATPase function (ATP1B1), and RNA splicing (TRA2B). All of the identified human host proteins interacted in yeast with NP from both human and swine origin. Proteins PAICS, ATP1B1, TRA2B, and SLC30A9 have been previously identified in studies related to host response to the IAV. Host proteins SLC30A9 and CKAP5 displayed the strongest interactions with IAV NP in yeast in a quantitative β-gal assay.

## Methods

### Strains, plasmids, media, microbial growth conditions, and reagents

Unless indicated otherwise, yeast strains (Table 3) were grown in liquid culture at 30°C at 180 rpm and on agar plates at 30°C. Bacterial strains were grown in liquid culture at 37°C at 200 rpm and on agar plates at 37°C. Selectable markers for the bait (pGBKT7) and prey (pGADT7) plasmids in the yeast *Saccharomyces cerevisiae* and bacterium *E. coli* are listed in Table 3. All media and Y2H reagents were purchased from Clontech (Mountain View, CA).

**Table 3:** **Strains and plasmids**

**Yeast strain**	**Genotype**	**Reporter genes**
Y2HGold	*MATa, trp1-901, leu2-3, 112, ura3-52, his3-200, gal4Δ, gal80Δ, LYS2::GAL1UAS–Gal1TATA–His3, GAL2UAS–Gal2TATA–Ade2 URA3::MEL1UAS–Mel1TATA, AUR1-C MEL1*	*ADE2, HIS3, MEL1, AUR1-C*
Y187	*ade2-101, trp1-901, leu2-3, 112, gal4Δ, gal80Δ, met–, URA3::GAL1UAS–Gal1TATA–LacZ, MEL1*	*MEL1, LacZ*
**Plasmid**	**Selectable markers (yeast, bacteria)**	**Additional information**
Plasmid pGBKT7 (bait vector)	*TRP1*, Kan	*GAL4(*1–147)DNA-BD, *TRP1*, *kanr*, c-Myc epitope tag
Plasmid pGADT7 (prey vector)	*LEU2*, Amp	*GAL4*(768–881)AD, *LEU2*, *ampr*, HA epitope tag

### Cloning Influenza A virus nucleoprotein into vector pGBKT7

The open reading frame (ORF) of the nucleoprotein gene from swine IAV H1N1 strain A/Sw/NC/44173/00 was amplified from the plasmid pScript A/Sw/NC/44173/00 NP (a gift from Christopher Olsen) by PCR using forward primer NP3F 5’-CATGGAGGCCGAATTCatggcgtctcaaggcaccaaacga-3’ and reverse primer NP2R 5’-GCAGGTCGACGGATCCattgtcatactcctctgcattgtctccgaaga-3’. The NP ORF from the human IAV H3N2 strain A/Ca/07/04 was amplified from plasmid pScript A/Ca/07/04 NP (a gift from Christopher Olsen) by PCR using forward primer NP2F 5’-CATGGAGGCCGAATTC atggcgtcccaaggcaccaaacg-3’ and reverse primer NP3R 5’-GCAGGTCGACGGATCC attgtcgtactcttctgcattgtctccgaaga-3’. For all primers the uppercase letters represent sequences homologous to the *Bam*HI-*Eco*RI-linearized vector pGBKT7, and lower case letters represent sequences specific for the NP ORF. Reactions included 200 ng plasmid template DNA, 1X Advantage HD Buffer (Clontech, Mountain View, CA), 0.2 mM dNTPs, 0.25 μM forward and reverse primers, and 0.625 Units Advantage HD Polymerase (Clontech, Mountain View, CA). Thermocycler conditions were as follows: 95°C for 3 minutes; 30 cycles of [95°C for 15 seconds, 55°C for 5 seconds, 72°C for 100 seconds], 72°C for 10 minutes.

InFusion cloning (Clontech, Mountain View, CA) was used to insert the NP ORFs into *BamHI*-*EcoRI*-linearized pGBKT7 3’ and in-frame with DNA encoding Gal4’s DNA binding domain (BD). Conditions were as follows: 60 ng gel-purified *Bam*HI-*Eco*RI-linearized pGBKT7, 50 ng gel-purified NP PCR product, and 1X InFusion HD Enzyme Premix in a 5 μl reaction were incubated at 50°C for 15 min. An aliquot of the cloning reaction was transformed into Stellar competent cells per the manufacturer’s directions (Clontech, Mountain View, CA). All plasmid sequences were verified by DNA sequencing.

### Yeast two-hybrid screen

The Yeast Two-Hybrid MatchMaker Gold system (Clontech, Mountain View, CA) was used to isolate prey plasmids that interact with bait pGBKT7-NP. A 50 ml SD-TRP liquid media culture inoculated with Y2HGold cells containing the bait plasmid pGBKT7-NP was grown in a 200 rpm shaker at 30 degrees to an OD600 of 0.8 (approximately 18 hours). Bait cells were pelleted by centrifugation, resuspended in 4 ml SD-TRP, and mixed with 45 ml YPDA in a 2 L flask with 2 × 10^7^ Y187 cells containing a commercially-available normalized HeLa S3 Mate and Plate cDNA prey library (Clontech, Mountain View, CA). Bait and prey cells were mated by slow shaking (40 rpm) for 24 hours at 30°C. After 20 hours, mating cells were observed microscopically for the presence of zygotes. Mated cells were centrifuged 1,000 × g for 10 min, and the pellet was resuspended in 5 ml 0.5X YPDA containing 50 μg/ml kanamycin. Mated cells (0.2 ml) were spread on each of 55 SD-LEU-TRP/X-a-Gal/125 ng/ml AbA agar plates, and plates were incubated 30°C for 5–8 days. This concentration of AbA is considered high stringency and could exclude isolation of low-affinity interactors. The mating efficiency was determined by plating 1:1,000 and 1:10,000 dilutions on SD-LEU, SD-TRP, and SD-LEU-TRP agar plates.

Blue colonies from the Y2H screen SD-LEU-TRP/X-a-Gal/AbA plates were picked as small patches to an SD-LEU-TRP plate, grown 2 days at 30°C, and replica plated to agar media (SD-ADE, SD-HIS, SD-LEU-TRP/X-a-Gal, SD-LEU-TRP/AbA) to test for activation of four reporter genes: *ADE2*, *HIS3*, *MEL1C*, and *AUR1-C*, respectively. Replica plates were incubated at 30°C and observed daily for 3 days. Cells from potential positive interactors (growing on all types of reporter media and blue/light blue on SD-LEU-TRP/X-α-Gal) were single-colony purified on SD-LEU-TRP/X-a-Gal and incubated at 30°C for 4 days. A single blue colony from each potential positive interaction strain was single-colony purified a second time on SD-LEU-TRP/X-α-Gal.

Following two rounds of streaking, the prey plasmid from each potential positive interaction yeast strain was isolated using the “Easy Yeast Plasmid Isolation Kit” (Clontech, Mountain View, CA). The resulting yeast plasmid DNAs were transformed into and subsequently re-isolated from *E. coli* cells.

### Testing potential positive prey plasmids for toxicity to yeast and autoactivation of the Y2H reporter genes

Isolated prey plasmid DNAs were re-introduced into Y2HGold cells using a yeast transformation system (Geno Technology). Y2HGold cells containing prey plasmids were single-colony purified on SD-Leu agar plates alongside a control (Y2HGold containing the empty prey vector pGADT7), and cell growth was observed after 3 days at 30C to test for toxicity. To test for false positives, patches of Y2HGold cells containing prey plasmids were replica plated to assess autoactivation of reporter genes on reporter media (SD-Ade, SD-His, SD-Leu-Trp + X-α-Gal, SD-Leu-Trp + AbA). Prey strains determined to be false positives in yeast were excluded from the pool of identified interacting host proteins.

### Bioinformatics

The 5’ and 3’ ends of human cDNA within each confirmed positive prey plasmid were determined by sequencing at the Iowa State University (ISU) DNA Facility using primers T7-1 (AATACGACTCACTATAG) and 3’AD (AGATGGTGCACGATGCACAG) or 3’ poly-TA (TTTTTTTTTTTTTTTTTTTTTTTTTA), respectively. The sequence data was used in a BLAST to query the NCBI human genome and human transcript databases.

### Maintenance of protein-protein interactions at 30°C and 37°C

Y2HGold yeast cells were separately co-transformed with a bait plasmid (either pGBKT7 bait vector containing NP from human influenza A strain H3N2, A/Ca/07/04 or NP from swine influenza A strain H1N1, A/Sw/NC/44173/00) and a prey plasmid (pGADT7 containing one of the human cDNAs isolated as a positive interactor with human NP bait). Y2HGold cells containing known bait/prey interactors pGBKT7-53 and pGADT7-T and known non-interactors pGBKT7-Lam and pGADT7-T were used as positive and negative controls, respectively. Additional negative interaction controls included Y2HGold cells containing human or swine NP (described above) and an empty prey vector pGADT7.

All transformants were grown in YPD media in overnight cultures and were diluted to a concentration of 5 × 10^6^ cells/ml (OD = 0.417). 200 ml of culture was placed into a 96 well plate. A 48 sample pinner was used to spot yeast onto 2 plates each of SD-Leu-Trp (control), and SD-His. One plate of each type was incubated at 30°C, the other was incubated at 37°C. Growth was assessed and images taken 4 days after the initial plating procedure. At least two replicates of each strain containing the bait (swine NP or human NP) and prey were tested on SD-Leu-Trp and SD-His at both 30°C and 37°C.

### ß-galactosidase assay

Diploid cells containing bait and prey were prepared by mating Y187 cells containing each of the prey plasmids with Y2HGold cells containing each of the bait plasmids (swine NP or human NP) on YPD agar (MatchMaker Gold Y2H protocol, Clontech, Mountainview, CA). Eight replicate samples of diploids were selected by subsequent growth on SD-Leu-Trp agar for each protein interaction. Diploid cells containing bait/prey were inoculated in 2 mL SD-Leu/Trp liquid media and grown in a 200 rpm shaker at 30°C overnight. 2 mL of the overnight cultures were diluted to 4 mL with YPD media and grown in a 200 rpm shaker at 30°C until they reached mid-log phase (OD_600_ of 0.5-0.8). Cultures were separated into 1.5 mL aliquots, pelleted, washed once, and then resuspended in 300 μL of Z buffer (16.1 g/L Na_2_HPO_4_⋅7H_2_O, 5.5 g/L NaH_2_PO_4_⋅H_2_O, 0.75 g/L KCl, 0.246 g/L MgSO_4_⋅7H_2_O, pH7.0). Samples of 100 μL were lysed by three freeze/thaw cycles with liquid nitrogen and a 37°C water bath. After the cells were lysed, the sample was split into two 150 μL aliquots. A blank tube, containing 100uL of Z buffer, was also set up. To analyze the enzyme activity, 350 μL of Z buffer with 0.27% β-mercaptoethanol and 4 mg/ml of ONPG as substrate were added. The yellow color was then allowed to develop at 30°C, and the reaction was stopped by the addition of 200 μL of 1 M Na_2_CO_3_. To remove cell debris, the samples were centrifuged for 10 minutes at 16,000 RCF, and OD_420_ values were recorded. In order to express the enzyme activity, Miller Units were calculated using the formula: 1000*OD_420_/(t*V*OD_600_) where t refers to the elapsed time, and V refers to the dilution factor (0.25 in this case).

## Abbreviations

Y2H: Yeast two-hybrid
SD-Leu: Synthetic defined media lacking leucine
SD-Trp: Synthetic defined media lacking tryptophan
SD-Leu-Trp: Synthetic defined media lacking leucine and tryptophan
SD-Ade: Synthetic defined media lacking adenine
SD-His: Synthetic defined media lacking histidine
IAV: Influenza A virus
AbA: Aureobasidin A
X-a-Gal: X-alpha galactose
YPDA: Yeast peptone dextrose adenine
amp: Ampicillin
kan: Kanamycin
NP: Nucleoprotein
BD: Binding domain
AD: Activation domain
ONPG: o-nitrophenyl β-d-glucopyranoside
